# Role of an imbalanced miRNAs axis in pathogenesis of psoriasis: novel perspectives based on review of the literature

**DOI:** 10.18632/oncotarget.12534

**Published:** 2016-10-08

**Authors:** Mao-Jie Wang, Yong-Yue Xu, Run-Yue Huang, Xiu-Min Chen, Hai-Ming Chen, Ling Han, Yu-Hong Yan, Chuan-Jian Lu

**Affiliations:** ^1^ The Second Affiliated Hospital, Guangzhou University of Chinese Medicine (Guangdong Provincial Hospital of Chinese Medicine), Guangzhou, China; ^2^ Section of Metabolic Diseases Research, University Medical Centre Utrecht, Utrecht University, Utrecht, The Netherlands; ^3^ Guangdong Provincial Key Laboratory of Clinical Research on Traditional Chinese Medicine Syndrome, Guangzhou, China

**Keywords:** psoriasis, imbalanced miRNAs axis, cell proliferation, cell differentiation, keratinocytes

## Abstract

**BACKGROUND:**

Specific profile of microRNAs (miRNAs, miR) expressed in psoriasis has been identified in the past few years, while the studies on roles and molecular mechanisms of these miRNAs are still on the way. In our previous study, four specific miRNAs (miR-31, miR-203, hsa-miR-99a and miR-125b) were found to be specifically altered in psoriatic lesions.We therefore conducted a systematic literature review in this study to reveal the role of these miRNAs in the pathogenesis of psoriasis in order to inform future research.

**METHODS:**

The related articles indexed in PubMed (MEDLINE) database were searched and analyzed. We identified eligible studies related to the mechanism research of miR-31, miR-203, hsa-miR-99a and miR-125b in psoriasis or psoriatic lesional skin from inception up to July 2016. The experts in the field of miRNAs and Psoriasis were involved in analysis process.

**RESULT:**

Both miR-31 and miR-203 are dramatically upregulated in psoriatic lesions. The former plays the pro-proliferative, pro-differentiative and pro-inflammatory roles and the latter holds the potentials for anti-proliferation, pro-inflammation and pro-differentiation in psoriatic keratinocytes. Conversely, both hsa-miR-99a and miR-125b are significantly downregulated in psoriatic skin. These two miRNAs are able to inhibit proliferation while promote differentiation of psoriatic keratinocytes, and miR-125b can also suppress inflammation in psoriatic lesions. By analyzing the contexts related to these miRNAs, we found that each of them does not act alone but rather work in concert with other miRNAs. The imbalance between miR-31/miR-203and hsa-miR-99a/miR-125b may contribute to the intense proliferation and abnormal differentiation of psoriatic keratinocytes, which is a characteristic of pathogenesis of psoriasis.

**CONCLUSION:**

An imbalanced miRNAs axis was for the first time outlined. Apparently, upregulation of miR-31/miR-203 and downregulation of hsa-miR-99a/miR-125b work together in concert to facilitate the development of psoriasis pathogenesis. Further work in this field holds the potentials to open a new way to study psoriasis.

## INTRODUCTION

Psoriasis is a chronic and complex inflammatory skin disorder with lesions displaying hyper-proliferative and abnormal differentiation. Psoriasis affects about 2-3% of population worldwide and causes negative consequences on the quality of life [1, 2]. Unfortunately, the treatment strategies for psoriasis are not effective sufficiently, which is, at least in part, due to that the molecular mechanisms underlying disease pathology are still largely unknown.It is now clear that microRNAs (miRNAs, miR) can regulate cell differentiation and proliferation, cytokine responses of keratinocytes, T-lymphocytes survival and activation, as well as the crosstalk between immunocytes and keratinocytes [3].The specific miRNAs profiles have been recently revealed in psoriasis [4],whereas the study for role and context of these miRNAs in psoriasis is still in its infancy.

Through a systematic review, we recently found that, among miRNAs dramatically altered in psoriasis, some candidates hold the potentials to be developed as the disease markers and therapeutic targets [5], especially four specific miRNAs, miR-31, miR-203, hsa-miR-99a and miR-125b. These four miRNAs caught our attention, since the imbalance expression between miR-31/miR203 and hsa-miR-99a/miR-125b may directly contribute to aberrant proliferation and differentiation in psoriasis. In the past, most of the studies put great efforts into mechanisms associated with one single miRNA, which leads to a lot of progress in understanding the pathogenesis of psoriasis. However, it is still difficult to understand disease pathogenesis in a systematical or comprehensive way. In this review, we discuss roles of these four specific miRNAs in pathogenesis of psoriasis and try to draw their intrinsic link in molecular mechanisms, as working on multiple molecules could be a better way to know systemic knowledge in psoriasis and thereby developing new strategies to treat this refractory disease in the future.

## RESULTS AND DISCUSSION

### Four key miRNAs were identified in psoriatic skin

Since the altered expression of miRNAs was for the first time determined in 2007, more and more cell- and region-specific miRNAs have been identified in psoriasis [1, 6–9]. However, the roles of miRNAs in psoriasis lesions have not been fully elucidated. In our last systemic review, we included any studies in which role of miRNAs in psoriasis was examined in relation to disease pathogenesis, diagnosis, and treatment. Therefore, we have identified the specific miRNAs profiles in human psoriatic skin, blood, and hair samples. Meanwhile, several unique miRNAs in inflammatory responses and immune dysfunction, as well as hyper-proliferative disorders of psoriatic lesions have been revealed [5]. Among these miRNAs, four miRNAs (miR-31, miR-203, hsa-miR-99a andmiR-125b) were dramatically and specifically altered in psoriatic skin and thereby exerting synergistic effects in increasing the severity of psoriasis. In order to reveal the mechanisms associated with these four miRNAs, we systematically reviewed and analyzed the related literatures in this study. The functions, target genes and the other related molecules of these four miRNAs were subsequently summarized in Table 1. The detailed information will be discussed below.

**Table 1:** The functions and related molecules of four key miRNAs identified in psoriasis skin

MiRNAs	Region, Tissues or Cells Expressed	Target Gene	Expression in Psoriasis	Roles of miRNAs in			Other Information	References
				Proliferation	Differentiation	Inflammation		
MiR-31	Psoriatic keratinocytes	ppp6c, STK40, FIH-1,EMP-1	Up	↑	↑	↑	MiR-31 can be induced by TGF-β1,IL-6 and IL-17A	[10–12, 16]
MiR-203	Keratinocytes in Psoriatic lesions; Normal human keratinocytes stimulated by combinations of pro-inflammatory cytokines	SOCS-3,p63, LASP1,RAPH1,RAN,TNFα, IL-24	Up		↑	↑	Skin-specific miRNA; Exclusively expressed in psoriatic keratinocytes	[6, 44, 50]
Hsa-miR-99a	Normal skin and psoriatic skin	IGF-1R	Down	↓	↑	-	One of the strongest down-regulated miRNAs in psoriatic skin	[9, 48]
MiR-125b	Fibroblasts, keratinocytes, and melanocytes	FGFR2, p63, Notch 1,TNFα	Down	↓	↑	↓	One of the most downregulated miRNAs in psoriasis skin.	[6, 15, 49]

ppp6c=protein phosphatase 6; STK40=serine/threonine kinase 40; FIH-1=factor-inhibiting hypoxiainducible factor 1; EMP-1=epithelial membrane protein 1; IGF-1R=insulin-like growth factor 1 receptor; FGFR2=fibroblast growth factor receptor 2; p63=protein 63; TNF=tumor necrosis factor; SOCS-3=suppressor of cytokine signaling 3; LASP1=LIM and SH3 domain protein 1; RAPH1=Ras-associated and pleckstrin homology domains-containing protein 1;RAN=Ras-related Nuclear protein; TGF-β=Transforming growth factor beta. “↑”=promote; “↓”=inhibit.

### MiR-31

MiR-31 expression is dramatically increased in psoriasis lesional skin, as compared to healthy skin and psoriasis nonlesional skin [10]. Further studies showed that in both psoriasis patients and psoriatic murine model, keratinocytes are major cell types responsible for the increased expression of miR-31 in psoriatic skin [10]. Moreover, miR-31 has been implicated as an important regulator of keratinocyte biology in psoriasis, e.g. inflammatory response, proliferation and differentiation [10–12]. In the current work, cytokines (tumor necrosis factor [TNF]-α, interleukin [IL]-22, transforming growth factor [TGF]-β1, IL-6, IL-20, interferon [IFN]-γ, and granulocyte-macrophage colony-stimulating factor [GM-CSF]), growth factors (epidermal growth factor and keratinocyte growth factor), and factors modulating cell differentiation were studied through the systematically review to see if they could regulate the expression of miR-31, the result turned out that only type 1 of transforming growth factor beta (TGF-β1) functions as a potent regulator of miR-31 in primary keratinocytes [10]. It is well known that TGF-β1 expression is increased in the epidermis and serum of psoriasis patients [13–15]. The overexpression of miR-31 triggered by TGF-β1 may be mostly through the activation of necrosis factor (NF)-κB, which is extensively involved in inflammation process including psoriatic pathogenesis [10]. In addition, another research found that IL-1α, IL-6, IL-17A, TNF-α, IFN-γ or IL-22 directly or indirectly activate the NF-kB signaling pathway, which further mediates miR-31 expression in keratinocytes [12]. Therefore, NF-κB is believed to have a crucial role in regulating miR-31 induction in psoriatic keratinocytes.

Moreover, protein phosphatase 6 (ppp6c) [12], serine/threonine kinase 40 (STK40) [10], factor-inhibiting hypoxia inducible factor 1 (FIH-1) [11], and epithelial membrane protein 1 (EMP-1) [16] have been reported as the direct target genes of miR-31. ppp6c, an inhibitor of the G1-S phase transition in the cell cycle, is diminished in human psoriatic epidermis. The inhibition of ppp6c is functionally essential for the biological effects mediated by miR-31, as evidenced by the fact that, by suppressing ppp6c, miR-31 contributes to basal keratinocyte proliferation and epidermal hyperplasia [12]. STK40 is another direct target of miR-31, and also it is a negative modulator of NF-κB signaling [10]. Therefore, possibly through inhibiting STK40, miR-31 activates NF-κB signaling, which further contributes to keratinocytes proliferation and maintains inflammation of psoriatic skin. With regard to FIH-1, it has been demonstrated that FIH-1 is markedly increased in psoriasis and can be suppressed by miR-31 *via* binding to the 3′ site of FIH-1 mRNA [11]. FIH-1 is able to promote keratinocyte proliferation [11], while on the other hand loss of FIH-1 promotes keratinocyte differentiation *via* a coordinate increase in Notch signaling [11], which is one of the key determinants controlling epithelial growth/differentiation [6, 17, 18]. Recently, a new research identified EMP-1 as a direct target of miR-31 in keratinocytes [16], which provides an explanation for a study showing that miR-31 unblocks the EMP-1-dependent repression of keratinocyte proliferation and migration.Collectively, these findings have established miR-31 as a multifunctional miRNA playing pro-proliferative, pro-differentiative and pro-inflammatory roles in epidermal keratinocytes.

### MiR-203

MiR-203 is the most abundant keratinocyte-specific miRNA in the epidermis [1], and it is highly and selectively expressed in mouse and human keratinocytes [19]. In fact, because of the ability to stop proliferation and block cell cycle at the G0/G1 phase, miR-203 is described as a ‘stem-ness’ repressor and an indirect promoter of differentiation process in epidermal keratinocytes [20]. The first reported target gene of miR-203 is suppressor of cytokine signaling 3 (SOCS-3), which is an important molecule in psoriatic plaque development [1, 21]. SOCS-3 is essentially involved in a negative feedback loop in cytokine signaling, and through this feedback loop STAT3 activation is suppressed in keratinocytes [22, 23]. Of note, STAT3 is Th17-related cytokines and positively correlated with expression of Th17 in psoriasis [22]. Apparently, therefore, the up-regulation of miR-203 leads to decreased SOCS-3 levels in psoriatic skin, which may consequently result in sustained activation of the STAT3 signaling pathway. Through this mechanism, miR-203 is able to resist the development of psoriatic plaques as well as contribute to increased/prolonged skin inflammation in response to T cell-derived cytokines.

Another target gene p63 can be specifically suppressed by miR-203 *via* binding to 3′-UTRs region of mRNA [20, 24]. As a member of the p53 family,p63 is able to regulate keratinocyte differentiation and epidermal stratification *via* the transactivation of the Notch ligand JAGGED-1 and the control of IKKα (IkB kinase-α) expression [25–32].Apart from SOCS-3 and p63, two other miR-203 target genes Ras-related Nuclear protein (RAN) and Ras-associated and pleckstrin homology domains-containing protein 1(RAPH1) has been recently identified [20, 33–35]. RAN is a member of the small GTP-binding protein (G-protein) superfamily and it was reported to be associated with cell proliferation and survival, nucleocytoplasmic transport and cytoskeletal dynamics [36–38]. RAPH1 is a regulator of actin dynamics taking part in cytoskeleton remodeling complexes [39]. Collectively, by analyzing functions of its target genes, the role of miR-203 in psoriatic keratinocytes can be interpreted as anti-proliferative, pro-inflammatory and pro-differentiative in psoriatic keratinocytes.

### Hsa-miR-99a

Hsa-miR-99a has been identified as a strongest down-regulated miRNA in psoriatic skin [9], while information about the role of this miRNA is limited. By bioinformatic prediction and experimental validation using microarray and polyribosomal loading analysis, Sun et al. identified that type 1 receptor of insulin-like growth factor (IGF-1R) is a direct target of hsa-miR-99a [40]. Elevation of IGF-1R enhances proliferation and inhibits differentiation in the psoriatic lesion [9], since IGF-1R can lead to activation of the Ras, Raf, mitogen-activated protein kinase (MAPK) and the phosphatidylinositol 3-kinase (PI3K) pathways [41]. Intriguingly, IGF-1R signaling can up-regulate the expression of hsa-miR-99a that in turn down-regulates the expression of IGF-1R, and by this procedure cell differentiation is facilitated [9]. Taken together, hsa-miR-99a is able to block cell proliferation and drive cells toward differentiation *via* specifically targeting IGF-1R in psoriatic skin.

### MiR-125b

MiR-125b is known to be expressed in normal skin and resident cells, such as fibroblasts, keratinocytes, and melanocytes [1]. By using microarray analysis, miR-125b was found to be significantly decreased in psoriasis skin in comparison with healthy skin [1]. Mechanism study has revealed that miR-125b suppresses psoriatic keratinocytes proliferation through inhibiting its directly target gene fibroblast growth factor receptor 2 (FGFR2), which is a receptor expressed on keratinocytes and upregulated in psoriatic lesion [15]. This study also showed that overexpression of miR-125b by transfecting with miR-125b precursor RNA in primary human keratinocytes not only suppressed cells proliferation but also induced the expression of several known differentiation markers. However, knocking down FGFR2 using siRNA methodcan only suppressed keratinocyte proliferation, but did not enhance differentiation, suggesting that the pro-differentiative effect of miR-125b is not related to FGFR2 [15].In addition to FGFR2, studies also identified p63 and Notch1 aspotent targets of miR-125b in epidermis cells. Both Notch1 and p63 are key molecules for regulatory signaling that controls keratinocyte differentiation and proliferation [27, 42]. According to these observations, it is reasonable to conclude that miR-125b holds a role to protect skin from psoriasis, which is in agreement with the downregulation of miR-125b in psoriasis skin.

### Imbalance miRNAs axis: miR-31/miR-203 and hsa-miR-99a/miR-125b

**Figure 1:**
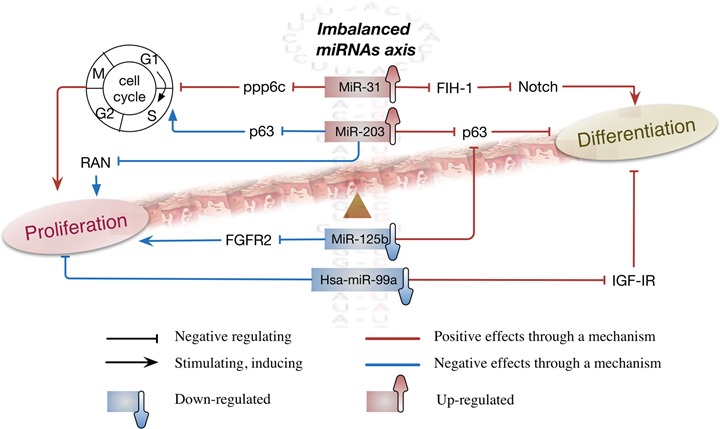
The imbalanced miRNAs axis consisted of upregulated pair of miR-31/miR-203 and down regulated pair of hsa-miR-99a/miR-125b plays a critical role in controlling proliferation and differentiation in psoriatic keratinocytes This imbalance between miR-31/miR-203 and hsa-miR-99a/miR-125b is like “Yin” and “Yang” in Chinese medicine, which means these two pairs of miRNAs are opposite or contrary in expression, but they are actually complementary, interconnected, and interdependent in the development of psoriasis pathogenesis. Abbreviations: ppp6c = protein phosphatase 6; FIH-1 = factor-inhibiting hypoxia-inducible factor 1; IGF-1R = insulin-like growth factor 1 receptor; FGFR2 = fibroblast growth factor receptor 2; p63 = protein 63; SOCS-3 = suppressor of cytokine signaling 3; PI3K = Phosphatidylinositol-4,5-bisphosphate 3-kinase; JAK = Janus Kinase; RAN = Ras-related Nuclear protein.

Taken together, we found that each of these miRNAs does not act alone but rather work in concert with other factors. Apparently, upregulation of miR-31/miR-203and downregulation of hsa-miR-99a/miR-125b work together in concert to form an imbalanced miRNAs axis, which contributes to the intense proliferation and abnormal differentiation of psoriatic keratinocytes through a signal network. This mechanism was depicted in Figure 1.

Briefly, in a synergistic manner, all of these four miRNAs contribute to cell differentiation by regulating the expression of FIH-1, p63 and IGF-IR. However, such a synergistic effect is weakened by decreased expression of hsa-miR-99a/miR-125b in psoriatic lesions. In addition, the downregulated pair of hsa-miR-99a/miR-125b plays a positive role in cell proliferation, which may cause imbalance between proliferation and differentiation of psoriatic keratinocytes, thereby contributing to psoriasis pathogenesis. Interesting, by targeting to ppp6c and p63 respectively, the upregulated pair of miR-31/miR-203 exert an opposite effect on cell cycle and then show pro-proliferation and anti-proliferation separately. Although these two miRNAs are positive in cell differentiation and contrary in cell proliferation, both can facilitate inflammation which is very important for the development of psoriasis pathogenesis. Therefore, undoubtedly, the imbalanced miRNAs axis, consisted of upregulated pair of miR-31/miR-203 and down regulated pair of hsa-miR-99a/miR-125b, plays a critical role in controlling proliferation and differentiation in psoriatic keratinocytes.

### Crosstalk between imbalanced miRNAs axis and key cytokines

**Figure 2:**
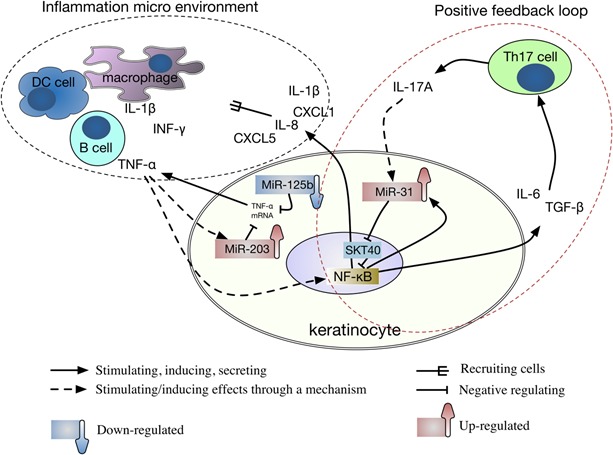
Crosstalk between imbalanced miRNAs axis and key cytokines Abbreviations: DC = Dendritic; Th17 = T helper type-17; STK40 = serine/threonine kinase 40; NF-κB = Nuclear factor kappa B; CXCLs = chemokine (C-X-C motif) ligands; IL = interleukin; TGF-β = Transforming growth factor beta; IFN-γ = Interferon gamma; TNF = tumor necrosis factor.

It is now widely accepted that close interdependence between keratinocytes and immune cells leads to epidermal pathological changes in psoriasis. In inflammatory microenvironment of psoriatic lesions, inflammatory factors, such as TNF-α, IL-17A and TGF-β, are secreted by keratinocytes and play a prominent role in recruitment and retention of inflammatory cells into psoriatic lesions [10, 43]. Immune cell-derived cytokines, in turn, act on keratinocytes to promote the expression of inflammatory genes which contribute to keratinocyte proliferation and impair keratinocyte differentiation. Importantly, crosstalk between miRNAs and inflammatory factors plays an important role in these biological processes. For example, TNF-α induces high expression of miR-203 [43], but unfortunately the increased miR-203 in turn inhibits TNF-α expression at transcriptional level [44]. The question is raised as to why both TNF-α and miR-203 are increased in psoriatic lesions? The imbalanced miRNAs axis could give an explanation (Figure 2). In contrast to miR-203, miR-125b is downregulated in psoriasis, which may partially bear responsibility for the elevated production of TNF-α, since miR-125b targets TNF-α directly at post-transcriptional level [45].

For crosstalk between miRNAs and inflammatory factors, we give another example as follows (Figure 2): In keratinocyte, TGF-β and IL-6 can induce miR-31 that is capable of inducing the production of IL-1β, chemokine (C-X-C motif) ligand 1 (CXCL1)/growth-related oncogene-a, CXCL5/epithelial-derived neutrophil-activating peptide 78, and CXCL8/IL-8 [10, 12].The mechanism for induction of miR-31 by TGF-βis recognized as being, at least in part, attributable tothe critical role of TGF-βin the differentiation of type 17 helper T-cells (Th17),which is known to play an essential pathogenic role in psoriasis [46]. IL-17A, the main cytokine produced by Th17 cells, is most effective in triggering downstream genes activation and directly or indirectly activates the NF-κB signaling pathway to promote miR-31 expression in keratinocytes [12]. Meanwhile, miR-31 directly targets STK40, which can inhibit TNF-induced NF-κB activation [13], thereby strengthening the IL-17A effect. Therefore, the crosstalk between inflammatory factors and miR-31 constitutes a positive feedback loop, which contributes to the persistence of inflammation micro environment in psoriatic lesions.

## CONCLUSIONS

Unlike other studies focusing on one single molecular, we studied the roles and intrinsic link of four miRNAs (miR-31, hsa-miR-99a, miR-125b and miR-203) that are dramatically and specifically altered in psoriatic skin in this work. The dramatical over-expression of miR-31 and miR-203 accompany with extreme downregulation of hsa-miR-99a and miR-125b form an imbalanced miRNAs axis, which plays a critical role in controlling proliferation and differentiation of psoriasis keratinocytes. In addition, the crosstalk between inflammatory factors and this imbalanced miRNAs axis is crucially involved in inflammation micro environment of psoriatic lesions, which further facilitates the development of psoriasis pathogenesis. This imbalance is like “Yin” and “Yang” in Chinese medicine, which means pairs of miR-31/miR-203 and hsa-miR-99a/miR-125b are opposite or contrary in expression, but they are actually complementary, interconnected, and interdependent in the development of psoriasis pathogenesis. Psoriasis is a complex and high heterogeneity disease. Therefore, further studies on this imbalanced miRNAs axis could be a better way to, not comprehensively but to some extent systemically, understand pathogenesis of psoriasis, and thereby developing new strategies to treat this refractory disease in the future.

## MATERIALS AND METHODS

**Figure 3:**
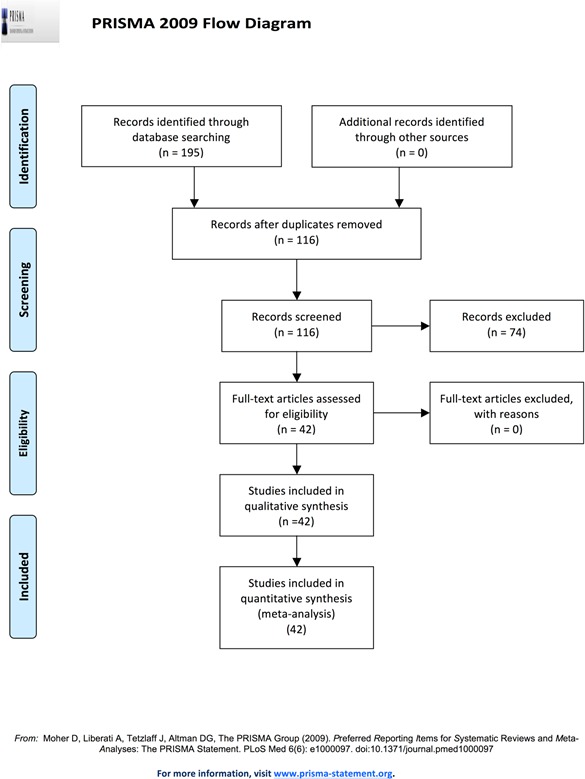
Methodology schematic for systematic review

This systematic review is reported according to the Preferred Reporting Items for Systematic Reviews and Meta- Analyses statement (PRISMA) (Figure 3) [14].

### Search methods

We searched the related articles indexed in PubMed (MEDLINE) database using Title/Abstract words ([“microRNA-31/99a/125b/203” or “;miR-31/99a/125b/203”]and [“psoriasis” or “keratinocyte” or “epithelium” or “skin”]) from inception up to July 2016.The references in articles selected were screened carefully. Therefore any researches matching the inclusion criteria in this study could be analyzed. The additional reports from the reference list of seminal reviews were also identified.There are no limitations impose on language, and three independent investigators conducted the searching process.

### Study selection

Inclusion criteria: All literature focusing on the mechanism of miR-31, miR-203, hsa-miR-99a and miR-125b were considered. No restriction for investigated subjects.In order to achieve the object, the reports including case report, review, meta-analysis and clinical trials were excluded. Three independent investigators conducted the searching process. Experts in the field of miRNAs and dermatology were involved in discussion and analyzing process.
